# Aging induced loss of complexity and dedifferentiation: consequences for coordination dynamics within and between brain, muscular and behavioral levels

**DOI:** 10.3389/fnagi.2014.00140

**Published:** 2014-06-27

**Authors:** Rita Sleimen-Malkoun, Jean-Jacques Temprado, S. Lee Hong

**Affiliations:** ^1^CNRS, Institut des Sciences du Mouvement UMR 7287, Aix-Marseille UniversitéMarseille, France; ^2^Inserm, Institut de Neurosciences des Systèmes UMR_S 1106, Faculté de Médecine Timone, Aix-Marseille UniversitéMarseille, France; ^3^Ohio Musculoskeletal and Neurological Institute, Ohio UniversityAthens, OH, USA

**Keywords:** aging, coordination dynamics, complexity, dedifferentiation, variability

## Abstract

Growing evidence demonstrates that aging not only leads to structural and functional alterations of individual components of the neuro-musculo-skeletal system (NMSS) but also results in a systemic re-organization of interactions within and between the different levels and functional domains. Understanding the principles that drive the dynamics of these re-organizations is an important challenge for aging research. The present *Hypothesis and Theory* paper is a contribution in this direction. We propose that age-related declines in brain and behavior that have been characterized in the literature as *dedifferentiation* and the *loss of complexity* (LOC) are: (i) synonymous; and (ii) integrated. We argue that a causal link between the aforementioned phenomena exists, evident in the dynamic changes occurring in the aging NMSS. Through models and methods provided by a dynamical systems approach to coordination processes in complex living systems, we: (i) formalize operational hypotheses about the general principles of changes in cross-level and cross-domain interactions during aging; and (ii) develop a theory of the aging NMSS based on the combination of the frameworks of coordination dynamics (CD), dedifferentiation, and LOC. Finally, we provide operational predictions in the study of aging at neural, muscular, and behavioral levels, which lead to testable hypotheses and an experimental agenda to explore the link between CD, LOC and dedifferentiation within and between these different levels.

## Introduction

Understanding the mechanisms underlying age-related declines across multiple functional subsystems ranks highly on the agenda of science and society. To achieve this objective, the most commonly adopted approach in aging research has emphasized dividing the neuro-musculo-skeletal system (NMSS) into smaller and presumably, more tractable units. From this perspective, declines in neural, cognitive, sensori-motor and muscular functioning are generally considered as separate domains, each having its own evolution over time. During the last 30 years, this approach has considerably improved our understanding of how aging affects the different levels of observation and functional sub-systems of the organism. However, it has also made aging research a complicated intellectual puzzle, with pieces that do not necessarily fit together, hence limiting our understanding of the aging NMSS as a whole. Despite (or rather, because of) the proliferation of isolated theories and potential mechanisms operating at different levels (about 300 have been identified by Medvedev, 1990), aging research remains data rich and theoretically poor.

In contrast to classic research, a growing body of literature in both aging and biomedical research acknowledges the fact that the human NMSS is a complex system comprising many interacting (complex) component subsystems that are connected over a variety of different scales of space and time (Chauvet, 1995; Buchman, 1996; Yates, 2008). Accordingly, it is also becoming clear that aging is a “parallel distributed process”. which not only affects the structures and functions of the individual subsystems but also the interactions between them. These changes alter the range of behaviors that the system can achieve, leading to impairments in behavioral adaptability (Lipsitz, 2002; Vaillancourt and Newell, 2002; Newell et al., 2006; Hong and Rebec, 2012). To better understand the coordination/coupling processes that occur during aging, both within and between the different subsystems and their consequences, an integrated framework—inspired by system biology and/or dynamical systems approach—is required (Haken, 1983; Kelso, 1995; Yates, 2008). The goal of the current paper is to develop a conceptual framework inspired by dynamical systems analysis to understand the general principles of age-related reorganization of the NMSS and its consequences on brain, behavioral and muscular dynamics. In the following, firstly, we review the literature on two phenomena that characterize the aging NMSS, namely, *dedifferentiation* and *loss of complexity* (LOC). We argue in this respect that these phenomena could actually be closely related. Indeed, although they seem to refer to different facets of aging, they both reflect both systemic and systematic reorganizations in the NMSS. Then, using the theory of coordination dynamics (CD; Kelso, 1995, 2009), we attempt to explain how dedifferentiation and LOC affect variability of system outputs and patterns dynamics at the levels of brain, muscles and behavior. Finally, we present hypotheses and empirical predictions that could be tested experimentally.

## The dedifferentiation hypothesis

Dedifferentiation can be defined as “*a* *process by which structures, mechanisms of behavior that were specialized for a given function lose their specialization and become simplified, less distinct or common to different functions”* (modified from Baltes and Lindenberger, 1997). Historically, the concept of dedifferentiation was introduced by Baltes and colleagues (Baltes, 1980; Baltes et al., 1998) to account for age-related increases in the correlation between levels of performance on different cognitive tasks. Dedifferentiation suggests the existence of a common cause of cognitive declines in aging (e.g., a general slowing of information processing; Birren, 1965; Birren et al., 1980; Cerella, 1985, 1991, 1994; Bashore, 1994; Salthouse, 1996), arising from reduced distinctiveness of mental representations and/or increased neural noise (e.g., a deficit in catecholaminergic modulation; Li et al., 2001). Both cross-sectional and longitudinal studies show that performance on sensory, cognitive and motor tasks are more correlated in the elderly, supporting the existence of *cognitive-motor dedifferentiation* (Lindenberger and Baltes, 1994; Baltes and Lindenberger, 1997; Lindenberger and Ghisletta, 2009). The following sections review existing evidence on dedifferentiation in brain and muscles.

### Dedifferentiation in brain function

Numerous brain-imaging studies have shown that the aging brain accommodates anatomical and physiological changes by re-organizing activation patterns between neural ensembles (Cabeza, 2002; Reuter-Lorenz, 2002; Ward, 2006; Serrien et al., 2007; see Park and Reuter-Lorenz, 2009 for review and theoretical account; Seidler et al., 2010). Specifically, in addition to stronger activation in dedicated regions, older adults generally exhibit activation of additional areas of the brain not observed (or only marginally) in young participants. For instance, it has been shown that brain dedifferentiation manifests in a shift from unilateral to bilateral activation (Cabeza, 2002; Cabeza et al., 2002; Ward, 2006) and/or an increase in activation of prefrontal areas (Heuninckx et al., 2005, 2008; Serrien et al., 2007).

During motor tasks, dedifferentiation takes the form of an increase in activation of neural structures presumably dedicated to cognitive processes (Heuninckx et al., 2005, 2008; Serrien et al., 2007; Park and Reuter-Lorenz, 2009; Seidler et al., 2010). This expanded activation is generally more pronounced with increasing motor task complexity, presumably reflecting greater involvement of executive control processes (Mattay et al., 2002; Ward and Frackowiak, 2003; Heuninckx et al., 2005). This hypothesis is supported by dual-task studies that have shown cognitive permeation of the motor domain i.e., interdependencies between sensorimotor and cognitive processes, becomes accentuated during aging (Li and Lindenberger, 2002; Schäfer et al., 2006; Schaefer and Schumacher, 2010). Dedifferentiated activation is also not limited to performance-based contexts. Comparable effects of aging have also been described during learning (i.e., dedifferentiation between explicit and implicit learning; Dennis and Cabeza, 2011), visual processing (i.e., dedifferentiation between pathways involved in faces, places and objects recognition; Park et al., 2004) and memory functions (i.e., between episodic and working memory; Papenberg et al., 2011).

### Dedifferentiation in muscle structure and function

One of the primary effects of aging on human musculature is a change in muscle fiber composition. Specifically, as reviewed in Lexell (1995), and recently demonstrated by Nilwik et al. (2013), the loss of muscle mass in aging (i.e., sarcopenia) is dominated by declines in the size of the fast-twitch fibers. While young adults have a nearly even ratio of fast- (type II) and slow (type I)-twitch muscle fibers, the elderly exhibit a higher proportion of slow-twitch fibers (see Table 2 from Lexell, 1995, for a summary). In addition, apart from atrophy of the type II fibers, aging also results in “clustering” or “grouping” of muscle fibers. In the young, fast- and slow-twitch fibers are almost evenly distributed or scattered in a muscle cross-section. With aging, muscle fibers form clusters as type I fibers form visibly distinct groups from type II fibers (see Andersen, 2003 for a review).

While not often discussed, it is important to note that “hybrid” muscle fibers also exist (see Pette and Staron, 2000, for a review), leading to altered contractile properties that fall between exclusively type I and type II fibers (Hilber et al., 1999). Aging leads to an increase in the proportion of hybrid fibers within a muscle (Monemi et al., 1999; Pette and Staron, 2000). These morphological changes would lead to dedifferentiation in muscle function, as aged muscles will exhibit a high level of homogeneity in contractile rate and force generation capacity. Effectively, as muscle fiber structure and function is homogenized, their ability to contract at different speeds and generate different force levels is restricted, hence narrowing their functional range. The dedifferentiation in muscle structure and function would leave them in a state where they are limited to acting on narrower scales of space and time.

## Loss of complexity

The LOC hypothesis was introduced 30 years ago in biomedical research by the pioneering work of Lipsitz and Goldberger (1992) on heart rate variability (HRV). Using nonlinear time series analysis (i.e., approximate entropy, ApEn but see Costa et al., 2002; Peng et al., 2009; Bravi et al., 2011, for reviews of the different methods), these authors observed a tendency toward more regular fluctuations in HRV (i.e., “less complex patterns” of variability) during aging and disease, which remained undetected by variance-based measures (coefficient of variation, SD). These changes have been interpreted as a LOC, which is currently considered as a generic driving principle of aging in a wide range of functional systems. LOC has even been hypothesized to be an indicator of the transition from normal aging to frailty (Lipsitz, 2002, 2004; Lang et al., 2009). However, increased behavioral variability is widely viewed as a hallmark of aging (Hultsch et al., 2008). Greater magnitudes of intra-individual variability in cognitive and motor performance are commonly attributed to increased levels of Gaussian noise produced at anatomical, functional and neuro-modulatory levels of the central nervous system (CNS; see MacDonald et al., 2009b for a review). However, there is further evidence that the magnitude and structure of variability may change independently of one another during aging (Slifkin and Newell, 1999; Sosnoff et al., 2006). This suggests that “variability” (amplitude) and “complexity” (pattern) of fluctuations stem from different origins and might have different functional significance (Sosnoff et al., 2006; McIntosh et al., 2010; Balasubramaniam and Torre, 2012).

The LOC hypothesis has been supported in studies of physiological, cognitive, and motor systems (see Vaillancourt and Newell, 2002; Newell et al., 2006; Rey-Robert et al., 2011). Yet, the effects of aging on the complexity of behavioral output fluctuations have been shown to depend on the functional system under investigation and/or the task being performed (Vaillancourt and Newell, 2002). This raised the proposal that aging impairs behavioral adaptability by restricting the ability to alter levels of behavioral complexity (Vaillancourt and Newell, 2002). Thus, a critical property of the system seems to be its capability to reorganize the interactions between its components (i.e., its functional degrees of freedom) to adjust the degree of unpredictability of behavioral fluctuations to meet task demands (Vaillancourt and Newell, 2002), leading to a proposal of the *Change in Complexity Hypothesis* by some of the authors of this article (CICH; Rey-Robert et al., 2011).

Changes in complexity have also been investigated in brain aging research. In resting state EEG studies where the subject is not engaged in any type of cognitive or motor task (i.e., under the instruction to relax), the elderly exhibit higher levels of brain signal complexity (Anokhin et al., 1996; Pierce et al., 2000, 2003; Müller and Lindenberger, 2012). Similar findings were also reported using fMRI (Yang et al., 2013). Conversely, task-driven brain activation signals seem to express a smaller complexity reduction in older subjects in comparison to the young (Müller and Lindenberger, 2012). Additionally, age-related changes of brain signal complexity appear to be scale-dependent. Using Multiscale entropy (MSE), McIntosh et al. (2014) found that, the elderly possess less complex brain signals at coarse time-scales, and more complex signaling at fine time-scales in comparison to the young. fMRI studies show an age-related decrease in brain signal variance as measured by the standard deviation of BOLD activity, and this was reported in both resting and task-driven states (Garrett et al., 2011, 2013; however, see Yang et al., 2013 for a conflicting result).

Altogether these findings support the systemic nature of age-related changes in the complexity of behavioral and brain signals. However, the direction of these changes (increase versus decrease) and the in-between level mapping is not straightforward and deserves further investigation.

## Linking loss of complexity and dedifferentiation in the neuro-musculo-skeletal system

Although LOC and dedifferentiation hypotheses have developed independently in the literature, there is evidence to indicate that they constitute two intertwined facets of the same underlying aging process. The systemic breakdown of the structure of fluctuations of behavioral outputs observed during aging are currently attributed to changes in coupling interactions (i.e., functional synergies) between the components of the different system over multiple temporal and spatial scales (Lipsitz, 2002; Vaillancourt and Newell, 2002; Newell et al., 2006). A plausible hypothesis is that LOC might arise, at least in part, from dedifferentiation occurring within and between the different subsystems.

At this juncture, an integrative theoretical framework that connects the LOCH and dedifferentiation hypotheses is needed. Four critical domains must be accounted for, namely: (1) *tasks—*classification of constraints and metrics of behavioral difficulty; (2) *structures—*anatomical and biochemical changes from organ to molecular level; (3) *function—*coordination of the individual structures and levels of organization; and (4) *behavior—*overt measures of systems outputs (i.e., pattern dynamics and behavioral output fluctuations). In this respect, we contend that principles of self-organization in complex systems identified by physics/dynamical systems theory (e.g., Glass and Mackey, 1988; Lipsitz, 2002, 2004; West, 2006) are essential. As a step in this direction, Vaillancourt and Newell (2002) proposed a standard approach to infer changes in the complexity of a system (independent of its nature) and its consequences on behavioral state and output fluctuations. They argued that a system’s complexity depends on: (i) the number of independent variables that is needed to reproduce or predict the output of the system; (ii) the functional states of the different components; and (iii) noise present in the system. To our knowledge, no systematic exploration of the consequences of separate or concomitant changes in the different factors identified by Vaillancourt and Newell (2002) has been undertaken in the literature. To achieve this objective, the challenge is finding task protocols that are rich enough to capture the coupling mechanism and properties of the neuro-behavioral system, but not so complicated that it precludes modeling. We contend that it could be done through the use of the conceptual framework, task paradigms and the methods of analysis of *CD*.

## Coordination dynamics as a conceptual framework for the study of the NMSS

CD is a conceptual framework dedicated to the study of coordinative processes that occur within and between brain and behavioral levels in a wide range of tasks. It refers to set of principles developed to capture the formation and functional adaptation of synergies and coordination patterns to meet different demands (Kelso, 2009, 2012; Tognoli and Kelso, 2009). From this perspective, coupling and self-organization properties allow the adaptive assembly, stabilization and dismantling of synergies between functional components and subsystems. Coupling over multiple spatial and temporal scales also ensure efficient informational exchanges within the neurobehavioral system through feedback loops and regulation processes (Slifkin and Newell, 1999; West, 2006; West and Grigolini, 2010; McIntosh et al., 2014).

These coordinative processes are evident in the presence of multiple stable states in the neurobehavioral repertoire and the ability to switch between these stable states to adapt to task or environmental constraints. Whatever the level of observation or the system under consideration, pattern dynamics capture the time-evolution of collective variables characterizing the state of the system (order parameters) under the influence of a set of internal and external constraints of various origins (control parameters). These control parameters may trigger switching between the different spontaneous states of the system, without prescribing these states. At a more abstract level, CD can be conceived as an evolving landscape of “attractor” wells, which is best represented by a potential function (Haken, 1977, 1983): the deeper the wells of the landscape, the more stable the patterns and the more resistant these patterns will be to perturbations. Control parameters modulate the landscape of attractors, thereby leading to loss of stability of behavioral states and phase transitions between them.

### Behavioral coordination dynamics

**Figure 1:**
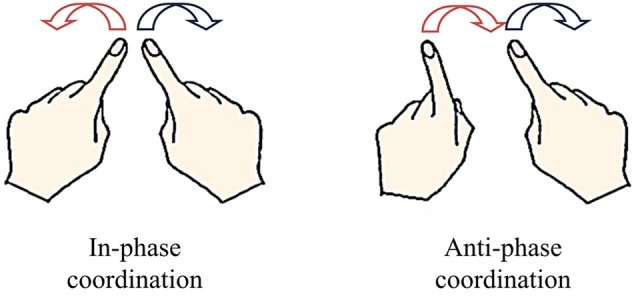
**Stable patterns of spontaneous bimanual coordination**. The in-phase pattern consists in symmetric movements in opposite directions (0° of relative phase) involving the simultaneous activation of homologous muscles. The anti-phase pattern consists in parallel movements in the same directions (180° of relative phase) involving the simultaneous activation of antagonist muscles.

One of the most representative paradigms of CD in living systems is that using rhythmic bimanual movements (Kelso et al., 1981; Kelso, 1984; Haken et al., 1985; see Kelso, 1995, for an overview). In this task, the relative phase between the displacements of each limb is considered as the collective variable (order parameter), which captures the dynamics of coordination patterns (i.e., their evolution over time) under the effects of constraints of various origins. The neurobehavioral repertoire is characterized by the presence of two preferred stable patterns of coordination: in-phase and anti-phase (Kelso, 1984), which can be spontaneously produced when participants are instructed to move their arms rhythmically in synergy. The in-phase pattern involves symmetric motion of the hands in opposite directions, whereas the anti-phase pattern involves motion in the same direction (Figure 1).

**Figure 2:**
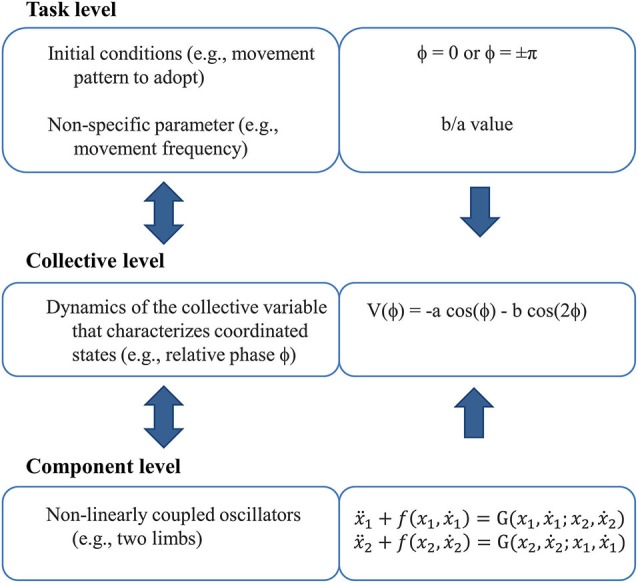
**The tripartite scheme (inspired from Kelso, 1995)**. It links the three phenomenological levels of description from the perspective of dynamic pattern theory.

Stability and flexibility are salient features of CD that have been elucidated by driving the bimanual system toward a point of instability where a *phase transition* subsequently occurs (i.e., an abrupt switch in the order parameter). The anti-phase pattern is generally considered to be less stable than the in-phase pattern, as a spontaneous switch from the former to the latter occurs when oscillation frequency (control parameter) increased beyond a given critical value (Kelso, 1981, 1984). Phase transitions are preceded by a destabilization of the current pattern (anti-phase), resulting from decrease in coupling strength, assuming the presence of noise of constant magnitude (Schöner et al., 1986), evidenced by a dramatic increase in relative phase fluctuations. Such “critical fluctuations” decrease following the phase transition, once the in-phase pattern is adopted (Kelso et al., 1986). These spontaneous/intrinsic dynamics of bimanual coordination are formalized through a tripartite scheme (Figure 2) in which the dynamics of the relative phase arise from a low-energy (nonlinear) coupling function linking nonlinear oscillators that represent the limbs (Haken et al., 1985; see Kelso, 1995, 2009, for detailed developments).

The CD formalization of attractor landscape and pattern dynamics is not limited to spontaneous dynamics of bimanual coordination patterns. For one, it has been extended to the study of different movement tasks (e.g., Kelso et al., 1990; Bardy et al., 1999; Temprado and Laurent, 2004; Salesse and Temprado, 2005; Sleimen-Malkoun et al., 2012) and in different populations (e.g., Temprado et al., 2010; Sleimen-Malkoun et al., 2011, 2013). In addition, it has been applied to the study of how the spontaneous dynamics is shaped through cognitive factors as attention (Temprado et al., 1999; Monno et al., 2002), intention (Scholz and Kelso, 1990), and learning (Zanone and Kelso, 1992), sometimes termed “directed or goal-directed dynamics”. Here, intention, attention and learning provide behavioral information that forms a continuous “force” to shape the dynamics of the collective variable. Intentionally switching from one pattern to another is an example in this regard (Scholz and Kelso, 1990). Selective attentional focus on an existing pattern (e.g., in-phase or anti-phase) generates behavioral information that does not compete with intrinsic tendencies, increases the stability of the coordination pattern (Lee et al., 1996; Temprado et al., 1999) and delays or even prevents the phase transition from occurring. In the case of learning, behavioral information may be perceptually specified by metronomes or memory, leading to greater stability of a previously unstable coordination pattern (Yamanishi et al., 1980; Zanone and Kelso, 1992). Although the information is different in both cases, visual feedback versus memorized information, similar modifications to the dynamics of bimanual coordination have been found (e.g., Schöner et al., 1992).

### Coordination dynamics in brain and behavior

**Figure 3:**
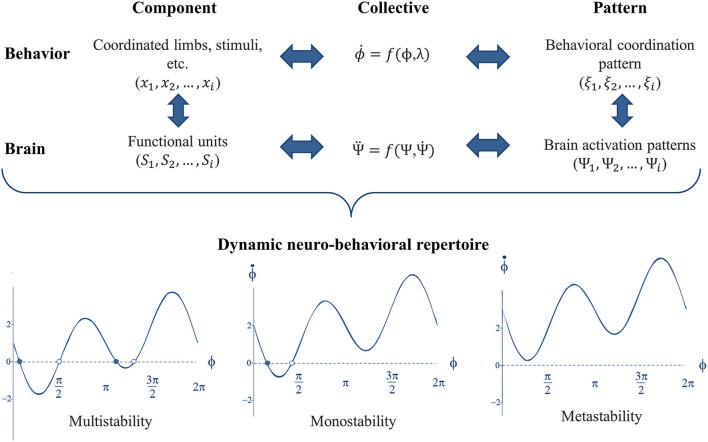
**Traversing scales of the neuro-behavioral system by virtue of shared dynamics**. At each level of organization, e.g., brain and behavior, the complex dynamics is expressed through lower dimensional patterns that can be captured through the dynamics of the collective variable(s). Accordingly, the neurobehavioral system possesses a dynamic repertoire of attractor states, in which multiple stable attractors can coexist (multistability) or not (monostability). Attractors can also be virtually present as “ghost attractors” giving rise to both phase trapping and phase scattering dynamical flows (metastability).

The presence and the switching between multiple patterns of activity across neural ensembles have now been shown in the brain (Meyer-Lindenberg et al., 2002; Jantzen et al., 2009; see Fuchs and Jirsa, 2008, and Kelso, 2009, for reviews). Subsequently, a multi-level approach was developed to connect phenomenological findings at the behavioral level to underlying neural mechanisms (see Jirsa and Haken, 1996; Jirsa et al., 1998; Kelso et al., 1999, 2013). To that aim, functionally relevant (coupled) components were identified at the brain level as it was previously done for the behavioral level. Fuchs et al. (2000) proposed a model to capture the relationship between rhythmic finger movements and neuronal activation. The model accounts for the presence of two stable states at low movement frequencies and predicts the destabilization of the anti-phase at higher frequencies. Thus, CD allowed traversing the different scales of the neuro-behavioral system to connect neural and behavioral dynamics (see Kelso et al., 2013, for a recent overview), presented in Figure 3.

### Coordination dynamics at muscular level

Whether and how neuro-muscular factors have a role in neuro-behavioral CD has led to considerable debate over the last decade (Carson et al., 2000; Mechsner et al., 2001; Temprado et al., 2003; Carson and Kelso, 2004; Mechsner, 2004). Initially (Kelso, 1981, 1984), phase transitions reflected a switch from simultaneous activation of non-homologous muscle groups (flexors-extensors) to activation of homologous muscles (flexor-flexor/extensor-extensor). These observations have been extended to wrist and upper limb oscillations, suggesting that the “default” output of brain CD was the activation of homologous muscles, forming the core of inter-limb CD in a wide range of different tasks.

However, a number of subsequent experiments have demonstrated that the intrinsic dynamics of behavioral patterns were relatively independent of neuro-muscular factors. For instance, in ipsilateral hand-foot coordination, Baldissera et al. (1982, 1991) showed that phase transition prominently depended on directional coupling, instead of muscular synergies. This hypothesis was confirmed in inter-personal coordination tasks: transitions from movements performed in opposite directions to patterns of iso-directional movements were systematically observed (e.g., Schmidt et al., 1990; Temprado and Laurent, 2004). These findings were interpreted as evidence supporting task-dependent informational coupling at the brain level. Mechsner et al. (2001) challenged the *neuro-muscular hypothesis* in bimanual finger coordination by showing that the bias of two-finger oscillations was towards perceptually-based spatial symmetry, irrespective of the muscles involved. Accordingly, they suggested that spontaneous bimanual coordination phenomena were perceptually driven (see also Mechsner, 2004). These debates about the role of intrinsic muscular synergies in inter-limb CD (e.g., Carson, 2004; Carson and Kelso, 2004), eventually converged on a consensus that behavioral CD result from a coalition of (task-dependent) constraints of various origins, including those arising from neuro-muscular factors (e.g., Temprado et al., 2003; Temprado and Salesse, 2004; Salesse and Temprado, 2005).

## A coordination dynamics perspective on neuro-behavioral aging

The framework of CD has been scarcely applied in aging research (see Greene and Williams, 1996; Temprado et al., 2010; Sleimen-Malkoun et al., 2013, for noticeable exceptions). The above developments suggest however that it might constitute a conceptual framework to age-related adaptations of a complex neuro-behavioral system on fast and slow time-scales.

### Aging ^1^ as complex neuro-behavioral system dynamics

The description of the time-evolution of the whole neuro-behavioral system, which emerges from complex interactions, is a critical issue in aging research. Performance curves alone are not able to capture declines in behavioral adaptability as it demands the capacity to preserve two apparently contradictory properties, namely stability and flexibility. Stability is classically indexed by variability surrounding a behavioral pattern that is to be maintained and the ability to resist perturbations to this pattern. Flexibility is indexed by the number of intrinsic patterns in the repertoire (i.e., multi-stability) and the ability to switch between them (i.e., transitions). It should be noted that long-range correlations within a time-series are sometimes considered as an indirect marker of system flexibility (Lipsitz, 2002), although (to our knowledge) the empirical confirmation of this hypothesis through appropriate protocols (i.e., perturbation studies) is lacking in aging literature. Despite this lack of direct evidence, from a dynamical systems perspective, variability and pattern dynamics are markers of system’s adaptability that should also be able to serve as indices of functional status in the aging NMSS.

### The dynamics of functional status during aging

**Figure 4:**
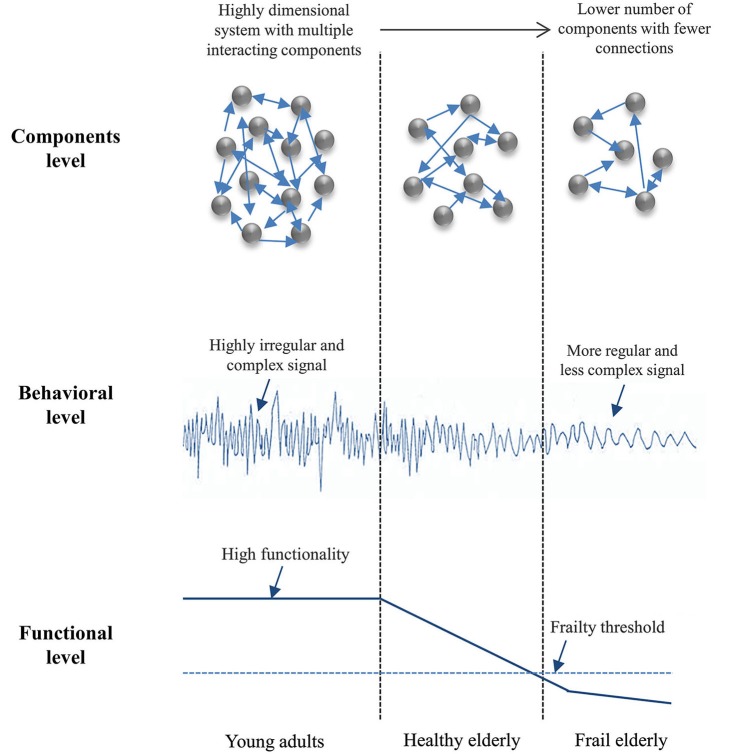
**Loss of complexity with age and frailty (inspired from Lipsitz, 2002)**. A representation of how the interacting sub-systems/components (system’s inputs) produce highly complex dynamics (system’s output) in young adults with high functionality, due to the richness of the underlying interactions; and as the number of components and the complexity of their interactions drop down, behavior becomes increasingly predictable and the organism loses its functionality (e.g., in elderly). The frailty threshold represents the critical level of function below which the organism can no longer adapt to stress.

Although it is currently admitted that age-related LOC of the entire neuro-behavioral system may lead to nonlinear changes in functional status over time (Goldberger et al., 2002), a precise description of these states is lacking in the literature. This issue refers to whether and how many “biological ages” can be distinguished on the basis of specific markers, independent of chronological age. Frailty—a geriatric syndrome associated with increased vulnerability, higher rate of morbidity and loss of autonomy—might be heuristic in this respect. Indeed, frailty is viewed as the signature of the degradation of multiple subsystems that normally contribute, through their (weak) couplings, to flexible behavioral adaptations to stressors of various origins (see Clegg et al., 2013; Cesari et al., 2014, for overviews). Even if this general definition is widely accepted in the literature, true system views of frailty are scarce and there are few issues that remain a matter of debate, namely: (i) how frailty can be characterized systemically, if not as the sum of declines in individual parts; and (ii) how changes in levels of systemic (dys) function are detected during healthy aging, at the onset of frailty, and during its progression. Lipsitz (2002, 2004) hypothesized that frailty might be reflected in a global loss of physiological complexity (Figure 4). Unfortunately, there is no absolute measure of optimal complexity (i.e., only relative change is informative) and, consequently, precluding a definition of a critical threshold that determines the transition to frailty (e.g., Lipsitz, 2002, 2004; Vaillancourt and Newell, 2002; Newell et al., 2006).

The empirical focus on physiological or behavioral output complexity, however, did not provide concrete framework to describe the age-related changes in coordination processes operating within and between the different subsystems. Thus, at least, the general characterizations of functional status through variability/complexity analysis (i.e., long range correlated structures), at a system level, should be complemented by a precise description of the time-evolution of the behavioral repertoire during aging. In the ensuing sections we describe how pattern dynamics, as a marker of evolving stability and flexibility, evolve in aging.

### The dynamics of intrinsic patterns of the behavioral repertoire during aging

**Figure 5:**
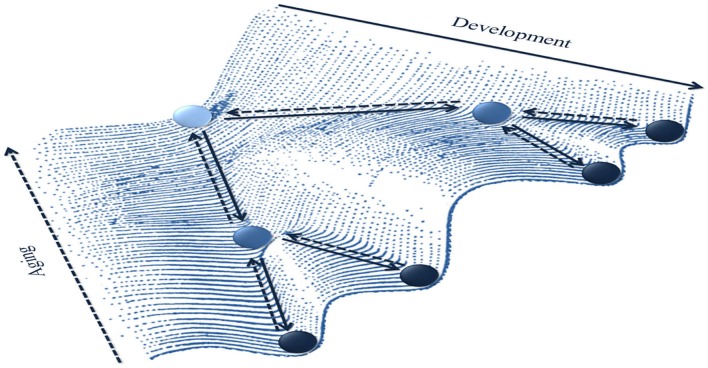
**Aging as a macro dynamic phenomenon on an epigenetic landscape (inspired by Waddington, 1942)**. A metaphoric representation of the life-span evolution of the landscape of behavioral attractors. During development, the number of attractor stable states (represented by wells in which a rolling ball can be trapped) increases through a differentiation process (filled lines arrows). Conversely, during aging (dashed lines arrows), the landscape goes through a dedifferentiation process leading to the disappearance or merging of some attractors.

At a general level, the aging NMSS can be addressed by studying: (i) the emergence of new patterns; (ii) the stabilization, destabilization and transitions between existing patterns; or even (iii) the loss of patterns of the behavioral repertoire. Landscapes of behavioral attractors can be used to represent these macroscopic dynamics (Figure 5). For instance, the epigenetic landscape initially introduced by Waddington (1942, 1957) has been subsequently popularized in development literature by Muchisky et al. (1996), and is now used in numerous domains (e.g., Aimetti, 2009; see Baedke (2013), for overview and discussion).

These landscapes allow one to envision the expression of aging in cognitive-motor skills (posture, locomotion, object manipulation, inter-limb coordination, etc.) as evolving behavioral (attractor) states. An increase in the number of wells represents the enrichment of the repertoire through a differentiation process, which can be formalized as pitchfork bifurcations. Smith and Thelen (2003) elegantly laid the ground for this idea by characterizing behavioral dynamics during development as a landscape with wells of different depths, i.e., varying stability (see also Newell et al., 2003, 2005). The framework of developmental epigenetic landscape can be extended to lifespan to account for emerging, modifying and dissolving behavioral patterns during aging as a result of the coalition of multiple (i.e., genetic, chemical, cellular, structural, environmental) underlying factors. A decrease in the number of wells can be considered a signature of dedifferentiation that could result from saddle-node bifurcations (old wells cease to exist by dead-ending) or from merging with another well.

Taking bimanual coordination as a representative illustration, we can envision age-related changes from the Haken-Kelso-Bunz model (HKB) potential function. We introduce the dimension of time (i.e., chronological age taken as a control parameter) to represent the deformation of the HKB potential landscape that mimics the changes in the spontaneous dynamics of bimanual patterns. At the most basic level of the HKB model: (i) only two terms are used, thus, only in-phase and anti-phase wells are present; and (ii) the attractor strengths are scaled in proportion to one another. What this means is that the healthy landscape comprises stronger and weaker attractors whose attractiveness is scaled in proportion to one another. Age can be hypothesized to drag the system to function in the middle where all wells tend to have lower and progressively more similar levels of attractiveness. The landscape would be dedifferentiated as all of the attractors would lose their scale-invariance, leading to greater similarity in strength as the highs and lows are no longer present. The result is a more evenly distributed landscape with attractors of equal or near-equal strength.

The complete dedifferentiation at the level of the collective variables (i.e., attractors on the landscape) would mean that the subcomponents are de-coupled from one another as any dynamic relationship between any of them would be equally viable, leading to increased magnitude of variability. Correspondingly, the individual subcomponents would then return to their natural oscillations or intrinsic dynamics (Haken et al., 1985), or even cease to oscillate (Daido, 2008), leading to a LOC. At the most general level, such a deformation of the HKB model would represent a system in which the potential energy is not conserved, since the tradeoff between potential and the number of attractor wells is broken.^2^ As Zanone and Kelso (1997) have demonstrated (in conjunction with the inherent properties of the HKB model), a solution for maintaining potential energy constant in the system is strengthening an existing attractor wells, at the expense of the other well. This perspective of aging-induced loss of multistability affords a different account from the dedifferentiation perspective, which was discussed above. Indeed, one could expect that during aging some attractors become deeper and stronger. Such changes would come at the expense of reduced strength at the other attractors or even their disappearance. This assumption is consistent with recent studies by some of us, in which we provided empirical evidence of age-related change in attractor landscape, namely, a decrease in pattern stability (Temprado et al., 2010) and a loss of existing patterns (Sleimen-Malkoun et al., 2013). From this perspective, one can envision the deformation of the HKB model as if the subject started with a higher level of *b/a* ratio and the decrease of lower, critical values. However, in the HKB model, *b/a* supposedly mimics the effect of frequency on coupling strength at task-related time scale. Consequently, the longer time dimension of aging should be rather added under the form of another extension that changes the initial stability of intrinsic patterns, decrease their stability over time or even leads to loss of attractor states (see Newell et al., 2008 for modeling the inverse tendency during development).

Age-related deformation of the HKB potential landscape that represents the dynamics of intrinsic patterns presumably influences the directed dynamics that is, how the behavioral repertoire is shaped, temporarily or durably, through cognitive factors as attention, intention and learning in older adults. This issue is of particular importance for aging research, according to the cognitive-motor dedifferentiation that occurs over time.

## Linking variability and complexity of neuro-behavioral outputs to pattern dynamics: an entry point for understanding age-related reorganization in the NMSS

At this point in the paper, the question remains of how pattern dynamics give rise to the observed NMSS declines in aging. In addition, the role of noise must also be incorporated in the conceptualization which is frequently considered to be an explanation for increased variability in cognitive and motor tasks in aging (see MacDonald et al., 2009a). Empirically, attempts to describe changes in system’s output variability (including structured fluctuations) and pattern dynamics using the same task have been scarce. In the following, we offer a dynamical system reading/interpretation of studies of relevance to this issue at behavioral, brain and muscular levels, and that, in both young and aged NMSS. We also point out the directions that must be explored in future work.

### Variability and the dynamics of behavioral patterns

Originally, fluctuations in rhythmic behavior were considered to be a product of random events (Wing and Kristofferson, 1973). Pioneering studies demonstrated long-range correlations structure of fluctuations of coordination patterns in finger tapping tasks (i.e., syncopation and synchronization, Chen et al., 1997; Ding et al., 2002) exhibiting that fluctuations in the tapping pattern were not random. In this type of task, participants must flex their index finger “on-” (synchronization) or “off-” a metronome beat (syncopation), at different frequencies. The synchronization (corresponding to in-phase pattern) and syncopation (i.e., anti-phase) patterns have been shown to follow the same dynamics as those described in bimanual coordination (see modeling efforts in Kelso et al., 1990; Kelso, 1995). In these experiments, the authors showed that correlated structures of fluctuations: (i) were different between syncopation and synchronization patterns; and (ii) depended on the cognitive strategy used to perform the task (e.g., finger extension on the beat instead of flexion off).

Torre and collaborators (Torre et al., 2007; Torre, 2010) extended these studies from one finger to bimanual coordination (see also Schmidt et al., 1991). They hypothesized that these fluctuations are indicative of the compromise between stability and flexibility of the neuro-behavioral system and thus, play a specific role in pattern dynamics. Their results provided evidence for 1/*f* noise in both in-phase and anti-phase patterns and showed a greater presence of long-range correlations in relative phase fluctuations were negatively correlated with the critical frequency of transition in young adults (Torre et al., 2007). From a LOC perspective, participants with more complex relative fluctuations were able to delay the phase transition to a higher critical frequency. In contrast, no relation was observed between the amplitude of variability (as measured by the signal variance) and critical frequency. This result suggests different and specific functional roles for structure and amplitude of variability.

Torre and Balasubramaniam (Torre and Balasubramaniam, 2011; Balasubramaniam and Torre, 2012) tested the potential relationship between magnitude and pattern of variability in bimanual coordination tasks. Their reasoning was that a positive correlation between magnitude and pattern of variability would create a “snowball effect”, pushing relative phase away from its current value. Conversely, a negative correlation would have a conservative effect, thereby maintaining the pattern within its current boundaries. Their results confirmed this hypothesis as a negative correlation between amplitude and structure of variability was observed for the anti-phase pattern at high frequencies. However, how these phenomena are related to mechanisms underlying pattern dynamics (coupling strength, time delays, component-pattern relationship…) remains to be determined. Moreover, aging consequences have never been investigated in this context. One would suspect that if aging reduces the complexity of coordinated fluctuations, it would also reduce the maximal frequency at which a given pattern can be sustained.

### Variability and the dynamics of brain activity

As it was recently argued by some of us (Hong and Rebec, 2012), variability and noise in brain activity serve a functional role (Ghosh et al., 2008; Deco et al., 2009, 2011; Garrett et al., 2011), making the ability to “shift” and distribute noise around the brain essential. Effectively, the healthy brain is able to modulate noise and variability depending on task demands and desired behavioral output (Hong and Rebec, 2012).

Network model studies showed how the interplay between brain structural connectivity, noise level and interaction delays shapes the functional dynamics (Ghosh et al., 2008; Deco et al., 2011). Specifically, they showed that neuro-anatomical connectivity gives rise to a dynamic multistable attractor landscape that is functionally relevant. Indeed, even in the absence of any task and any external stimulation (i.e., the resting-state of the brain), brain activity shows structured spatio-temporal patterns characterized by a latent multistable dynamics. As a critical property, the healthy brain is continuously exploring its dynamic repertoire of attractor states without getting trapped in a single state. In the brain aging literature, the most investigated aspect of the dynamics is related to brain signal fluctuations, their magnitude, structure and distribution over cerebral areas. Despite a number of discrepancies in available literature (e.g., in terms of used methods, task conditions and reported results), some guiding principles can still be extracted.

One recurrent observation is an inverted relation between age-related changes in resting-state and in task-evoked activity. Specifically, whereas at rest EEG signals show more complex fluctuations in older than in younger adults (i.e., less deterministic and with higher dimensionality—Anokhin et al., 1996; Pierce et al., 2000, 2003; Müller and Lindenberger, 2012), during task-relevant activity the tendency seems to be reversed: MSE decreases for coarse scales, as well as distributed entropy (McIntosh et al., 2014), whereas dimensional complexity reduction during task has a tendency to attenuate (Müller and Lindenberger, 2012). However, McIntosh et al. (2014) revealed the presence of temporal-scale dependency, according to which complexity values were higher in elderly at fine time-scales. On slow-time scales (i.e., in fMRI studies), brain activity was found to be less variable in elderly, in both fixation (no task) and different cognitive tasks (Garrett et al., 2011, 2013), with a greater increase from fixation to task in younger adults (Garrett et al., 2013). Specifically, young participants exhibited higher variability in 84% of brain areas, whereas, in the remaining 16% (mainly cerebellum and sub-cortical structures) older subjects were the more variable (Garrett et al., 2011).

In a similar vein, McIntosh et al. (2014) found that multi-scale entropy at fine scales (taken as an indicator of the amount of information processed locally) increased with aging, whereas distributed entropy (i.e., mutual information shared by two sources and linked to functional connectivity) and complexity at coarse time-scales decreased. Aging, in this context, leads to the spatial clustering of information processing, instead of transmitting information across the brain like the young subjects. Pierce et al. (2000, 2003) found that older subjects displayed a higher complexity of spatial distribution of EEG activity suggesting a decrease in the degree of coordination among cortical areas in the aged-brain. Garrett et al. (2011) showed that the elderly exhibited nearly indistinguishable levels of variability across brain structures (i.e., spatial dedifferentiation) while young adults had a 78% difference between the less (mainly sub-cortical) and more variable (cortical) structures. Interestingly, young subjects appear to modulate the magnitude and the spatial representation of the variability of their brain activation to a greater extent and in a more expansive set in region than older subjects do (Garrett et al., 2013). These findings converge to indicate that aging leads to a reduction in coordinated activity. The elderly seem to rely on localized information processing in a manner that is similar to the clustering of different muscle fiber types.

### Variability and the interaction between brain and behavioral levels

Age-related increases in behavioral variability are currently considered to reflect the amount of neural noise, that is, the age-related increase in random background of activity in the CNS (Li et al., 2000; Li and Sikström, 2002). The presence of continually fluctuating background activity, random or not, is pervasive at all levels of the CNS, even at the most molecular level (see Faisal et al., 2008). Based on widely accepted declines in dopaminergic neurotransmission with aging, neuro-computational models predict a greater variability in neural signaling, and as a consequence, greater behavioral variability and loss of distinctiveness of mental representations (Li et al., 2001). Still, there is debate regarding whether there is truly random (white) noise in the NMSS (Sosnoff and Newell, 2011). This debate, however, is only pertinent if one assumes that neural noise must be: (i) white (i.e., flat power spectrum); and (ii) truly random and uncorrelated. Actually, the activity of neurons *in-vitro* is correlated, following a power law distribution known as the avalanche dynamic (Beggs and Plenz, 2003). Moreover, the relationship between dopamine and neural dynamics takes on an inverted U-shape, where correlations within the signal (i.e., lowest noise or least randomness) are achieved at the mid-range of dopamine levels (Stewart and Plenz, 2006). Either too much or too little dopamine leads to a breakdown in the internal correlations and a flattening of the neural activity distribution, which has consequence of reduced precision and consistency of the produced behavior, based on Li et al. (2000) computational model.

Whether brain signal variability could be taken as a performance predictor is yet another unsettled debate. A strong evidence for a positive correlation was provided by Garrett et al. (2011) by measuring SD of BOLD activity in young and older subjects in three cognitive tasks. In this study, the authors found that brain variability was highly correlated with age and performance: younger, faster (i.e., shorter reaction times) and more consistent (i.e., lower intra-individual standard deviations) participants exhibited greater levels of brain signal variability. Using similar task conditions, McIntosh et al. (2014) reported scale-dependent differences in brain signal (recorded with EEG and MEG) complexity between young and older participants, who displayed comparable accuracy but slower reaction times. Nevertheless, no direct correlation analyses were performed between behavioral (performance) and entropy (brain complexity) measures. In other studies, also investigating brain variability in the context of perceptual and cognitive tasks, the relation between brain fluctuations and performance was even less clear. For instance, Müller and Lindenberger (2012) found associations between perceptual speed performance and brain dynamics for only a few EEG electrodes. Another example can be seen in the Pierce et al. (2000) study in which significant correlations were found between complexity in the spatial distribution over time of EEG activity and only one measure amongst the twelve measures provided by the Visual and Auditory Continuous Performance Test. In particular, higher algorithmic complexity was associated with higher scores on the Consistency-Visual measure. It should be noted, however, that in this specific context, older adults performed significantly better than younger adults.

Strikingly, in all of the reviewed studies, none investigated movement tasks, thereby raising a number of open questions, which should be addressed in future research. One issue of great interest is the systematic exploration of age-related changes in magnitude and structure of fluctuations in both brain and behavior, along with the respective correspondences. A preliminary step would be determining how amplitude and structure of fluctuations relate to meta-stability/self-organizing criticality of activation patterns in the brain (Kelso, 2012). This constitutes an exciting entry point to explore whether and how the aging modifies of the functional relationship between amplitude and structure of variability first within the brain then, between the different levels of organization such as brain and behavior, but also muscular activation.

### Variability and the dynamics of muscular activity

It is striking that in most studies on brain-behavior relationships, the dynamics of muscular activity is omitted. However, since aging leads to significant structural and functional changes in the nervous system (NS), at both central and peripheral levels, muscular function should be subsequently affected (Manini et al., 2013).

Most studies carried out to explore age-related changes in muscular activity focused on the consequences of peripheral modifications on the variability of force production.^3^ In this respect, structural changes in muscle composition are consistent with the findings of a LOC in force output in the elderly (see Morrison and Newell, 2012 for a recent review). Supposedly, the loss of range in muscle fiber contractility leads to a decreased number of time-scales along which force output can be modulated, hence, a less complex force output. With a reduction in capacity to alter force output on different time-scales and force amplitudes, the motor behavior of the elderly consistent with the LOCH would be expected. The literature is replete with evidence of narrowed functional ranges in motor behavior, where the LOC is observed across a variety of different functions, including gait, posture, tremor, and muscle force output (see Morrison and Newell, 2012 for a recent review). These findings support the hypothesis that there are commonalities between LOC and dedifferentiation.

Despite this, there is far less empirical evidence on the LOC in the musculature beyond motor behavior, that is, in terms of motor unit activity. Thus, experiments should be conducted to explore systematically the links between dedifferentiation, LOC in the musculature and behavioral dynamics (i.e., force outputs). As we reviewed earlier, one of the consequences of aging is a change in muscle fiber proportions, where there is a transition toward a greater proportion of slower, hybrid fibers. The consequence of dedifferentiation at this level would be to reduce the number of different time-scales of muscle contraction. Consequently, a decline in the ability to generate high and low forces (and consequently, fast and slow movements) is to be expected. Smoothness at high speeds would become difficult to achieve, as the necessary bell-shaped velocity profiles (see Harris and Wolpert, 1998) require large muscle forces both to initiate and terminate the movement. At low speeds (e.g., tai chi), jerky movements would still occur as all of the muscle contractions are occurring at a single rate. Thus, instead of a single, continuous movement arising from a combination of fast and slow contractions, the aged muscle would be restricted to a sequence of smaller movements, resulting in a jerky action. This phenomenon is identical to finding that the elderly have a reduced ability to generate smooth sinusoidal isometric force traces and difficulty in generating rapid corrections while attempting to maintain a constant force output (Vaillancourt and Newell, 2002).

Age-related alterations of central processes may also affect how the CNS generates patterns of muscle synergies (Carson, 2006) that is, how the large number of degrees of freedom of the musculoskeletal system is mastered to achieve goal-directed tasks (Bernstein, 1967). In this respect, the coordination problem encountered by the aging CNS at neuro-muscular level is fundamentally a dimensional reduction problem, consisting of the mapping of an infinite number of different task goals onto an infinite set of muscle patterns. A current efficient solution envisaged in the literature is that pre-assembled muscular synergies would be represented in the CNS under the form of a small set of discrete, time-varying muscle synergies, which are combined to generate muscle patterns (d’Avella and Tresch, 2002; d’Avella et al., 2003; Tresch and Jarc, 2009; Dominici et al., 2011). As a general adaptation principle, these synergies would be scaled in amplitude and time to achieve flexible goals in a wide variety of motor tasks (see d’Avella et al., 2003, for details). If one accepts this hypothesis, the question arises of how age-related dedifferentiation and LOC in the CNS: (i) modifies the repertoire of pre-assembled muscular synergies; and (ii) affects the scaling process, thereby impairing movement adaptability.

## Hypotheses and experimental agenda

As developed above, using CD affords an unique opportunity to connect, within an integrative approach, the LOC and dedifferentiation hypotheses by focusing on uncovering the effects of aging on complementary aspects of the NMSS (i.e., variability and pattern dynamics) and studying functional ranges rather than single dimensions of brain, muscles and behavior, leading to the following hypotheses:

*A. Aging leads to a generalized intra-individual LOC and dedifferentiation in the different functional subsystems*. The LOC hypothesis arises from separate studies carried out with different groups of participants. Thus, an intra-individual comparison of changes in complexity occurring across the different physiological, cognitive and motor systems is lacking in the literature. The hypothesis of intra-individual LOC could be tested by measuring intra-individual variability (including complexity indexes) of behavioral outputs of cognitive, sensori-motor, physiologic, neural, etc. systems in specific tasks (reaction time, force control, postural control, gait, etc.). One would expect to observe a convergence in behavior complexity in older participants across all of the subsystems being examined. Such general tendency would represent a dedifferentiation of the different subsystems, which leads to a less complex NMSS overall. An interesting avenue would be to explore conditions under which young and older adults differentially modulate their levels of behavioral and brain signal complexity in response to task constraints. Indeed, the modulation of the levels of complexity (depending on the task and the environment) presumably reflects the ability of the NMSS to manage its multiple of degrees of freedom in an optimal manner. Experimentally, it requires testing the subjects under: (i) spontaneous or minimally constrained conditions, in which the system can freely express its complex dynamics (e.g., resting state, postural tremor, oscillation at a natural frequency); and (ii) complex task constraints requiring the system to significantly reduce its fluctuations (e.g., cognitive-motor task-evoked activity, constrained trajectory, force production levels, or frequency).

*B. Information transmission underlying the control of cognitive-motor tasks is altered as a result of age-related LOC of the neurobehavioral system*. To explore this hypothesis, one could exploit the Complexity Matching Principle (West and Grigolini, 2010), which predicts that when several coupled sub-systems exchange information within a complex system to perform a task, the most efficient information transmission occurs when complexity is optimal. This hypothesis could be tested through the use of a dual-task situation associating a force control paradigm and a RT task. In this type of situation, in addition to the complexity of force fluctuations, one can assess the efficiency and cost of information processing by specific variables (i.e., signal to noise ratio and RT, respectively, Slifkin and Newell, 1999). At a preferred level of force production, one expects to observe the highest level of complexity, associated with optimal information processing, a high signal to noise ratio and a low RT.

Another hypothesis is that when initially independent systems with different levels of output complexity, are coupled, the system with a lower output complexity will move toward the one generating the higher level of complexity (Stepp and Turvey, 2010; Marmelat and Delignières, 2012). This hypothesis could be tested in an inter-personal coordination task (see Temprado and Laurent, 2004; Oullier and Kelso, 2009; Riley et al., 2011) by comparing: (i) young-young; (ii) old-old; and (iii) young-old pairs of participants. One predicts that limb components would modify their individual dynamics when assembled/coupled to produce a stable synergy. Specifically, when young and older participants are paired, informational coupling should allow for a transfer of complexity from higher (young adult) to lower (old). Thus, the motor output of the elderly will be more complex when performed in tandem with the young if compared against a solo performance of a similar motor task. If affirmative, it would have theoretical and practical implications for the use dyadic inter-generational associations in complex motor tasks as a mode of motor rehabilitation.

*C. As a result of dimensional reduction (i.e., decrease in functional degrees of freedom), aging leads to a narrowing the neurobehavioral repertoire and reduced adaptability to task demands*. At the very general level of epigenetic landscape, one should observe a decrease in the number of behavioral patterns in the repertoire. To limit the analysis to a single dimension, one could examine the deformation of behavioral landscape using a bimanual task protocol (i.e., the scanning paradigm; Zanone and Kelso, 1992, 1997). In case of an age-related loss of multistability, destabilization of one of the existing states is expected, hence deforming the behavioral landscape toward monostability. The state to-be lost will exhibit a flatter potential, i.e., lower stability. Empirically, these changes should be observable in elderly through an increase in behavioral variability (measured by relative phase variability) and attentional cost (measured by RT in a dual-task) associated with the anti-phase pattern, as well as more frequent transitions toward the in-phase pattern starting at rather low movement frequencies. Conversely, if aging modifies the landscape towards greater similarity between attractors, coordination patterns will exhibit similar levels of stability and behavior would be more easily locked into a single pattern. Changes in the structure of behavioral fluctuations would also be expected revealing a loss of criticality in the system, i.e., an inability to seamlessly transition between behavioral states.

As a result of the modification of intrinsic dynamics of the behavioral repertoire, one also predicts to observe increasing difficulties in learning new behavioral patterns. To test this hypothesis, one could explore whether phase transitions from anti-phase to in-phase can be delayed by extensive practice that is, by stabilization of an already existing pattern in the intrinsic repertoire. One could also explore whether phase transitions from anti-phase to in-phase can be delayed by extensive practice that is, by stabilizing an already existing pattern. This issue is important since it might show that older adults are able to preserve adaptability of their repertoire of cognitive-motor skills through appropriate training procedures. It would also be of interest to explore whether learning capacity depends on the route adopted to acquire and stabilize a new pattern (Kostrubiec et al., 2012).

The loss of adaptability that would be observed at the behavioral level should be mirrored in brain dynamics and in brain signal variability. Accordingly, several issues should be investigated using brain activity recordings (fMRI, EEG, MEG): (i) changes in complexity at different spatial and temporal scales; and (ii) loss of multistability in the aged-dynamic repertoire; (iii) changes in terms of coupling strength; (iv) expression of metastability; and (v) the relative contribution of local and global connectivity.

*D. Aging reduces the ability to resist and recover from perturbations*. An example of the reduced capacity to recovery from perturbations in the elderly is the cardiovascular response to the tilt-table (Lipsitz, 2002). Similar tests can be conducted using sensory or mechanical perturbations to test this hypothesis in motor behavior. Transcranial magnetic stimulation provides an opportunity to perturb the CNS, allowing the effects on neural communication to be measured using EEG, for example. Based on this hypothesis, it would be predicted that the elderly: (i) are more easily displaced from a given coordination pattern; and (ii) require more time to recover their original pattern following the perturbation.

*E. Dedifferentiation is accompanied by LOC in individual cognitive and sensorimotor functions and assembling of specific cognitive-motor synergies*. Here, the hypothesis is that age-related changes in cognitive-motor interplay (dedifferentiation) should modify the assembling of synergies associating cognitive and sensori-motor processes. In addition, cognitive-motor synergies should allow preserving the internal complexity of the whole cognitive-motor systems (and its adaptability), while the complexity of individual cognitive/sensorimotor outputs should decrease. This hypothesis could be tested using classic dual-task paradigms (see Temprado et al., 1999; Pellecchia et al., 2005). Such experiments would contribute to the current and still unsettled debate about the adaptive/non adaptive function of cognitive-motor dedifferentiation (e.g., Mattay et al., 2002; Heuninckx et al., 2008). Specifically, they will permit the determination of whether increased coupling between cognitive and motor processes allows preserving the complexity of the whole cognitive-motor system by association of functional structures (Chauvet, 1995). Experiments could also be carried out to study the effects of aging on intentional switching from in-phase to anti-phase and vice-versa. One can predict that switching time would be influenced by age due to the differential strength of coupling between limb components.

*F. The repertoire of pre-assembled muscular synergies should be reduced, thereby impairing movement adaptability*. To our knowledge, these issues have not been addressed in the literature until now. In this context, it can be predicted that: (i) in each muscle system, the repertoire of synergies should be reduced toward intrinsic synergies or even a subset of intrinsic synergies, thereby limiting flexible adaptation of movements (i.e., similar to dedifferentiation); and (ii) central and peripheral changes that are at origin of loss of muscular strength (i.e., dynapenia/sarcopenia) should strongly constrain the assembling of synergies within which the muscles are activated. How changes in variability (amplitude and structure) of behavioral outputs (kinetic and kinematic) reflect the modifications of these coordinative processes underlying the formation of muscle synergies remain to be further explored and modeled.

*G. Aging affects the interactions between behavioral dynamics, variability and complexity, and underlying large-scale neural network mechanisms*. CD in brain and behavior have been previously studied using MEG (e.g., Kelso et al., 1992; Fuchs et al., 2000) and EEG (Wallenstein et al., 1995; Mayville et al., 1999; Banerjee et al., 2012). However, to our knowledge, the effects of aging on the re-organization of cortical networks underlying stable behavioral patterns, the resulting instabilities, and phase transitions that occur under changes in control parameter have never been explored. In particular, the question arises as to: (i) whether maintaining bimanual coordination patterns is associated with more recruitment of additional networks to those that control unimanual movements instead of simply a temporal modulation of these unimanual networks; and (ii) what are the consequences of such recruitment on correlated evolutions of variability/complexity at brain and behavioral levels. Although in young participants, maintaining stable bimanual patterns seems to result from temporal modulation (Banerjee et al., 2012), the hypothesis of additional recruitment in the elderly is consistent with the dedifferentiation hypothesis. It would reflect more age-related cognitive involvement in maintaining coordination patterns (Heuninckx et al., 2005, 2008). In addition, evidence supporting a small amount of additional recruitment at the vicinity of the transition from anti-phase to in-phase has been provided by Banerjee et al. (2012). One can hypothesize that such amount of recruitment could be larger in older adults.

The issue of correlated changes in variability/complexity indexes at brain and behavioral levels also deserves to be addressed. Indeed, it has been suggested that amplitude and structure of variability might play specific roles in behavioral pattern dynamics (see above). Thus, it should be determined how these two types of variability are correlated with those observed for pattern of neural activity. To our knowledge, no strict correspondences have been established yet between the amplitude and structure of variability in brain and behavior. Additionally, existing literature on aging is largely biased towards the study of cognitive performance. Indeed, sensori-motor aging is largely under-investigated, although the use of continuous movement tasks offers the possibility of conducting nonlinear analyses on time series recorded for both brain and behavioral levels.

## Summary and conclusion

The present paper offers an integrative theoretical framework that provides a broader perspective on the consequences of aging on coordination processes that occur within the NMSS. It affords a global picture of the age-related dimensional reduction of the neuro-behavioral system leading to loss of behavioral flexibility. This systems view diverges from the conventional viewpoint that aging is the sum of decline(s) in function(s) away from a youthful state. Instead, driven by complexity changes and dedifferentiation within and between the different functional subsystems, aging is viewed as a systemic re-organization of the whole NMSS. From this perspective, our general hypothesis is that the emergence of order within and between the different levels during aging obeys to general principles (LOC) and underlying mechanisms (dedifferentiation) independent of the level of observation (i.e., neural, muscular, behavioral). At a methodological level, variability and pattern dynamics are two key markers to follow the functional status of the NMSS during aging and frailty. Employing a systems approach to study aging, i.e., a view that transcends different sub-systems and functional domains, significantly adds to the currently dominant approach comprising the study of separate sub-systems. By understanding how many different components and their interactions change with the course of aging yields important insight into compensatory and adaptive processes that occur as we age. Such a level of understanding could lead to novel approaches to promote healthy aging and reduce levels of disability in the elderly.

## Conflict of interest statement

The authors declare that the research was conducted in the absence of any commercial or financial relationships that could be construed as a potential conflict of interest.

## Footnotes

^1^Aging currently refers to changes that occur over life span. Here we use it to refer to the period from adulthood to old chronological age.

^2^Indeed, the HKB model is founded on a Fourier series with a doubling process that increases the number of potential wells in the system. When the number of parameters is increased, the number of attractor wells increases, but their “attractiveness”, is decreased.

^3^The relationship between kinematic and kinetic fluctuations remains to be elucidated.
